# Application of New Molecular Probes in the Diagnosis and Treatment of Malignant Tumors

**DOI:** 10.3390/cancers15194752

**Published:** 2023-09-27

**Authors:** Dengfeng Cheng, Hui Lu

**Affiliations:** 1 Department of Nuclear Medicine, Zhongshan Hospital, Fudan University, Shanghai 200032, China; 2 Department of Orthopedics, The First Affiliated Hospital, Zhejiang University, Hangzhou 310003, China

Molecular probes, specialized tools or substances meticulously designed to bind to specific molecules or biomarkers within cells, tissues, or biological samples, play a pivotal role in various domains such as biomedical research, diagnostics, and medical treatments [1]. They are instrumental in the detection, study, and manipulation of precise molecular targets. Recent years have witnessed a remarkable surge in the development and application of advanced molecular probes, characterized by their heightened specificity and enhanced capabilities. One notable trend in the realm of molecular probes is the continuous refinement of their design and their heightening sensitivity. These probes can be engineered to exhibit a strong affinity for cancer-related biomarkers, including genetic mutations or protein overexpressions, making them invaluable in both the diagnosis and treatment of malignant cancer [2]. In this Special Issue, we focus on the indispensable role played by molecular probes in the diagnosis and treatment of malignant tumors. 

This editorial features a study by Zheng et al., highlighting the potential of molecular subtyping in guiding diagnosis and personalized treatment decisions for esophageal squamous cell carcinoma (ESCC). This study identified two distinct molecular subtypes of ESCC, S1 and S2, and linked them to the tumor microenvironment, distinct immune pathways, and clinical outcomes [3]. Ye et al. introduce an innovative molecular imaging probe, denoted as 99mTcHYNIC-ADH-1, after rigorous development. Their study provides an initial assessment of its diagnostic utility for monitoring drug resistance in non-small-cell lung cancer (NSCLC). The results illuminate the significant promise of 99mTc-HYNIC-ADH-1 as an effective tool for evaluating drug resistance in NSCLC, owing to its rapid synthesis, exceptional radiochemical purity, robust stability, high labeling efficiency, lack of purification requirement, and prompt blood clearance [1]. Moreover, Yao et al. provide a comprehensive review in this publication, shedding light on the current understanding of malignant peripheral nerve sheath tumors (MPNSTs) and offering insights into the latest consensus on diagnostic approaches and treatment strategies [4]. Numerous studies have yielded diverse FAP-targeted probes, ranging from antibodies to boronic acid-based inhibitor molecules. Among these, quinoline-based FAP inhibitors (FAPIs) have emerged as the most promising candidates for use as radiopharmaceuticals in FAPI PET/CT imaging. Dong et al. offer an overview encompassing the evolution and clinical deployment of FAPI PET/CT by focusing on the diagnostic and therapeutic implications across a spectrum of tumor types, while also considering the future potential and advancements in FAPI imaging [2]. A further study compares neoadjuvant chemotherapy followed by radiofrequency ablation with neoadjuvant chemotherapy followed by hepatectomy for treating colorectal liver metastases. Their findings reveal that patients undergoing neoadjuvant chemotherapy followed by radiofrequency ablation experience rapid recovery, fewer complications, and a lower progression rate [5]. Zhou et al. explore the clinical features, phenotypic markers, and outcomes of diffuse large B-cell lymphoma (DLBCL) in HIV-infected and HIV-uninfected Chinese patients. The results indicate that HIV-infected DLBCL patients exhibit distinct blood and phenotypic markers, with lower response rates and one-year survival rates, likely due to increased hematological complications and widespread organ involvement [6]. Also, another study in this Special Issue suggests that TFF1 and TFF2 may function as potential tumor suppressors in Gastric cancer (GC), offering valuable insights for predicting overall survival (OS) in GC patients. These findings unveil a promising therapeutic avenue, where targeting TFFs could hold potential benefits for GC treatment [7]. In addition, a comprehensive investigation conducted by Li et al. analyzes the actionable landscape within the largest Asian melanoma cohort. Notably, they pioneer the utilization of DNA and RNA sequential sequencing to underscore the clinical advantages it brings to a broader spectrum of melanoma patients. Remarkably, the findings reveal that as many as 11.7% of patients previously considered undruggable could now be identified as actionable through this innovative sequencing approach. Furthermore, the implementation of RNA-NGS substantially increases the proportion of druggable fusion events from 2.56% to an impressive 17.27% [8]. This Special Issue also includes a collective review of glioma, the most prevalent primary intracranial tumor. The review primarily explores the application of CRISPR/Cas9 in glioma therapy, including the induction of antigens, antibodies, or receptors for immunotherapeutic purposes, the identification of critical clinical prognosis targets, and investigations into glioma’s pathogenesis. Additionally, the article outlines the future research prospects of CRISPR/Cas9 in glioma treatment while highlighting potential opportunities and challenges in this domain [9]. In a recent study conducted by Cao et al., a pioneering transformer-based end-to-end deep learning model, MVI-TR, was introduced as substantial preoperative predictive utility for early-stage hepatocellular carcinoma patients [10]. Furthermore, an examination of recent research elucidating the involvement of WT1-associated protein (WTAP) in oncogenesis and its potential therapeutic implications was undertaken [11]. Another article reviews the current epidemiology and mechanisms of cardiac toxicity in pan-cancer therapies, stressing the importance of assessing relevant risk factors [12].

In this Special Issue, we have explored the evolving role of advanced molecular probes. These specialized tools have become increasingly precise and sensitive, aiding in the detection and manipulation of specific molecules within cells and tissues. The articles cover various diseases, highlighting how molecular probes are reshaping diagnosis, treatment, and therapeutic possibilities. Collaborations between scientists, healthcare experts, and technologists promise to drive continued progress in this field, offering new avenues for disease management and improved patient outcomes.

## References

[1] Ye Q., Liu Z., Zhang S., Wang G., Wen G., Dong M. (2023). Development of 99mTc-Hynic-Adh-1 Molecular Probe Specifically Targeting N-Cadherin and Its Preliminary Experimental Study in Monitoring Drug Resistance of Non-Small-Cell Lung Cancer. Cancers.

[2] Dong Y., Zhou H., Alhaskawi A., Wang Z., Lai J., Yao C., Liu Z., Hasan Abdullah Ezzi S., Goutham Kota V., Hasan Abdulla Hasan Abdulla M. (2023). The Superiority of Fibroblast Activation Protein Inhibitor (FAPI) PET/CT Versus FDG PET/CT in the Diagnosis of Various Malignancies. Cancers.

[3] Zheng Y., Gao Q., Su X., Xiao C., Yu B., Huang S., Sun Y., Wu S., Wo Y., Xu Q. (2022). Genome-Wide DNA Methylation and Gene Expression Profiling Characterizes Molecular Subtypes of Esophagus Squamous Cell Carcinoma for Predicting Patient Survival and Immunotherapy Efficacy. Cancers.

[4] Yao C., Zhou H., Dong Y., Alhaskawi A., Hasan Abdullah Ezzi S., Wang Z., Lai J., Goutham Kota V., Hasan Abdulla Hasan Abdulla M., Lu H. (2023). Malignant Peripheral Nerve Sheath Tumors: Latest Concepts in Disease Pathogenesis and Clinical Management. Cancers.

[5] Chen Y., Huang Y., Xu L., Wu J., Han F., Jiang H., Zheng P., Xu D., Zhang Y. (2022). Neoadjuvant Chemotherapy Followed by Radiofrequency Ablation May Be a New Treatment Modality for Colorectal Liver Metastasis: A Propensity Score Matching Comparative Study. Cancers.

[6] Zhou M., Cheng J., Zhao H., Yang M., Yu W., Qin J., Lang G., Tao R., Cao Q., Huang Y. (2022). Clinical Features, Phenotypic Markers and Outcomes of Diffuse Large B-Cell Lymphoma between HIV-Infected and HIV-Uninfected Chinese Patients. Cancers.

[7] Qian Z., Jiang Y., Shou C., Yu J., Huang D., Xie H., Zhou L., Chen D., Zheng S. (2022). Validation of the DNA Methylation Landscape of TFF1/TFF2 in Gastric Cancer. Cancers.

[8] Li Y., Wang B., Wang C., Zhao D., Liu Z., Niu Y., Wang X., Li W., Zhu J., Tao H. (2023). Genomic and Transcriptional Profiling of Chinese Melanoma Patients Enhanced Potentially Druggable Targets: A Multicenter Study. Cancers.

[9] Kang X., Wang Y., Liu P., Huang B., Zhou B., Lu S., Geng W., Tang H. (2023). Progresses, Challenges, and Prospects of CRISPR/Cas9 Gene-Editing in Glioma Studies. Cancers.

[10] Cao L., Wang Q., Hong J., Han Y., Zhang W., Zhong X., Che Y., Ma Y., Du K., Wu D. (2023). MVI-TR: A Transformer-Based Deep Learning Model with Contrast-Enhanced CT for Preoperative Prediction of Microvascular Invasion in Hepatocellular Carcinoma. Cancers.

[11] Ju G., Lei J., Cai S., Liu S., Yin X., Peng C. (2023). The Emerging, Multifaceted Role of WTAP in Cancer and Cancer Therapeutics. Cancers.

[12] Chen M., Xue J., Wang M., Yang J., Chen T. (2023). Cardiovascular Complications of Pan-Cancer Therapies: The Need for Cardio-Oncology. Cancers.

